# Association of Human Leukocyte Antigen Class I Polymorphism with Spontaneous Clearance of Hepatitis B Surface Antigen in Qidong Han Population

**DOI:** 10.1155/2013/145725

**Published:** 2013-11-13

**Authors:** Fengqin Miao, Hang Sun, Ning Pan, Jinhuan Xu, Jie Qiu, Yuqing Shen, Wei Xie, Jianqiong Zhang

**Affiliations:** ^1^Medical School, Ministry of Education, Southeast University, Nanjing, Jiangsu 210009, China; ^2^Key Laboratory of Developmental Genes and Human Disease, Ministry of Education, Southeast University, Nanjing, Jiangsu 210096, China; ^3^The Second Affiliated Hospital of Southeast University, Nanjing, Jiangsu 210008, China

## Abstract

*Aim*. To investigate whether HLA class I polymorphisms could influence the clearance of hepatitis B surface antigen (HBsAg) in Qidong Han population. *Methods*. We genotyped HLA-A, -B, and -C loci of 448 individuals with HBV persistent infection and 140 persons with spontaneous clearance of HBsAg by polymerase chain reaction with sequencing based typing (PCR/SBT). All the individuals were unrelated males enrolled from Qidong Han population and were followed up for 10 years. *Results*. The frequency of *HLA-A*∗*33:03:01G* was increased in persistent HBV infection group (*P* value is 0.028), while frequency of *HLA-B*∗*13:01:01G* was increased in HBsAg clearance group (*P* value is 0.0004). *Conclusion*. These findings suggested that the host HLA class I polymorphism is an important factor in determining the outcomes of HBV infection.

## 1. Introduction

Hepatitis B virus (HBV) infection is a major public health problem. Two billion people are expected to be infected with HBV during their lifetime and about 350 million are estimated to be chronic carriers [1, 2]. Nowadays, there are about 120 million HBV chronic carriers in China [3]. HBV infection can cause broad-spectrum diseases, including chronic hepatitis, liver cirrhosis, and hepatocellular carcinoma [4]. The mechanism of HBV clearance and pathogenesis is not yet clearly defined, but host genetic component is one of the critical contributors, which includes age, sex, immune response, and so forth [5–8]. Therefore, understanding the role of host genetic variability in the pathogenesis of HBV infection will help us to uncover the basis for this viral persistence.

Human leukocyte antigen (HLA) is an integral component of the immune response on which majority of host genetic studies have focused. Numerous reports described the associations of highly polymorphic HLA gene with the outcome of a wide range of infectious diseases. It is because antiviral cytotoxic T lymphocytes (CTLs) are believed to play a major role in eradication of infection by virtue of their capacity to identify and kill virus-infected cells through recognition of viral peptides presented by HLA class I molecules [9]. Thus, HLA molecules are considered to govern the pathology of disease, progression, or regression along with the viral and environmental factors.

The genes of the HLA locus are located on the short arm of chromosome 6. They are arranged in three clusters: class I, class II, and class III. Many previous studies have shown the associations of certain HLA class I genes with the course of HBV infection, but these associations are inconsistent even within the same population [10, 11]. Han is the largest ethnic group in China. Among Han resident areas, Qidong is a famous place in China where the habitants have high incidence of HBV infection. In this study, to determine whether the HLA I alleles are associated with resistance or susceptibility to HBV infection in Qidong Han people, we analyzed the polymorphism of HLA I alleles in individuals who had HBV persistent infection or those who had HBsAg clearance using high resolution sequencing technique.

## 2. Materials and Methods

### 2.1. Ethics Statement

The protocol was approved by the Ethics Committee of the Qidong Liver Cancer Institute, and all patients provided written, informed consent before enrollment.

### 2.2. Study Subjects

A total of 728 unrelated male individuals with hepatitis B surface antigen (HBsAg) positive in sera were enrolled in the Institute of Liver Cancer of Qidong in 1995. They were followed up for 10 years and tested for HBV infection again. In 2005, 588 individuals were included in this cohort because of loss to follow-up and exclusion of the patients who were treated with anti-HBV therapy. Among these 588 persons, 448 remained HBsAg positive (named HBV persistent infection group), while 140 were negative for HBsAg but positive for both anti-hepatitis B virus core antigen (HBc) and anti-HBsAg (named HBsAg clearance group). None of the subjects were positive for hepatitis C virus (HCV), hepatitis D virus (HDV), and human immunodeficiency virus (HIV) antibody. All of them are Han population.

### 2.3. Serologic and Virologic Tests

Plasma samples were obtained from all the subjects and stored at −20°C. Their serologic status with regard to HBsAg, anti-HBs, anti-HBc, hepatitis B virus e antigen (HBeAg), and anti-HBe was determined by enzyme-linked immunosorbent assay according to the manufacturer's instructions (Boehringer-Mannheim, Munich, Germany).

### 2.4. Extraction of Genomic DNA and HLA Allele Genotyping

Genomic DNA for each individual was extracted from peripheral blood mononuclear cells using the QIAamp DNA blood kit (Qiagen Inc., Chatsworth, CA). All DNA samples (100–200 ng/mL) were stored at 4°C (short term) or −20°C (long term) in TE buffer (10 mM Tris-HCl, pH 8.0, 2 mM ethylene diamine tetraacetic acid) and diluted to 10–12 ng/mL in PCR.

Genomic DNA was amplified by the standard HLA locus-specific primers [12]. According to the routine sequence-based typing (SBT) method, exons 2 and 3 were sequenced in forward and reverse directions using a 3730XL DNA analyzer (Applied Biosystems, Foster City, CA) and BigDye Terminator v3.1 Cycle Sequencing Ready Reaction Kit (Applied Biosystems). The sequences were then analyzed using the online software SBT Interface (MHC database; National Center for Biotechnology Information; http://www.ncbi.nlm.nih.gov/mhc/). All groups of HLA alleles that have identical nucleotide sequences across the exons encoding the peptide binding domains (exons 2 and 3 for HLA class I alleles) are designated by a code, such as A*33:03:01G, which essentially contains A*33:03:01, A*33:15, A*33:25, and so on (http://hla.alleles.org/alleles/g_groups.html).

### 2.5. Statistical Analysis

The allele frequency was calculated as the number of the allele divided by the double numbers of the total samples. The significance of allelic associations was determined either by chi-square test or by Fisher's exact test (when there were less than five subjects in a cell). In order to reduce the number of comparisons with inadequate power, only alleles with frequency not less than 3% (29 alleles in this population) were analyzed. The level of significance was adjusted for multiple testing using the Bonferroni correction. According to the Bonferroni correction, the frequency of HLA allele was considered to be statistically different only when uncorrected *P* value was less than Pc (0.05/29 = 0.0017). HLA genotypes with frequency not less than 3% were checked for the Hardy-Weinberg equilibrium using *χ* ^2^ test. Data were analyzed by SPSS 13.0 data analysis software package. Odds ratios (OR) and their 95% confidence intervals were calculated.

## 3. Results

### 3.1. Patient Characteristics

588 Qidong Han persons who were tested as HBsAg positive in 1995 were included in this study. 448 were classified as HBV persistent infection group as they remained HBsAg positive 10 years of later. The HBsAg clearance group includes the remaining 140 individuals who were negative for HBsAg but positive for both anti-HBc and anti-HBs after 10 years follow-up. Age distribution showed no statistical difference between the two groups.

### 3.2. Frequencies of HLA-A, HLA-B, and HLA-C Alleles in the Study Groups

A total of 31 HLA-A alleles, 54 HLA-B alleles, and 35 HLA-C alleles were detected in the two study groups (Tables 1–3). Among them, the frequencies of 29 alleles were higher than 3%. A*24:02:01G was the most frequent HLA-A allele (Table 1), while B*46:01:01G and B*40:01:01G were the most frequent HLA-B alleles (Table 2), and C*01:02:01G was the most frequent HLA-C allele (Table 3). Meanwhile, four new alleles (HLA-C*12:10:02, HLA-B*40:122, HLA-B*40:127, and HLA-B*55:49) were identified by our previous study from this cohort and named by the WHO Nomenclature Committee [13, 14]. HLA genotype frequencies were consistent with Hardy-Weinberg equilibrium (*P* > 0.05).

### 3.3. Comparison of Frequency of HLA-A, HLA-B, and HLA-C Alleles between the Persistent Infection and HBsAg Clearance Groups

To maintain statistical power, only those alleles with the frequencies not less than 3% were compared between the persistent infection and HBsAg clearance groups. The frequency of HLA-A*33:03:01G allele in the HBV persistent infection group was higher than that in the HBsAg clearance group (6.36% versus 2.86%, *P* = 0.025, *OR* = 2.31, 95% CI: 1.09–4.90), whereas the frequency of HLA-B*13 : 01 : 01G was higher in the HBsAg clearance group than that in the persistent group (8.57% versus 3.46%, *P* = 0.0004, *OR* = 2.62, 95% CI: 1.51–4.54). However, only the frequency of HLA-B*13 : 01 : 01G increased significantly in HBsAg clearance group after the Bonferroni correction.

## 4. Discussion

It is doubtless that host genetic factors are associated with the clinical outcome of different infectious diseases. The most-studied host factors that might affect the susceptibility to viral infection are HLA genes [15, 16]. Among HLA genes, HLA class I genes encode HLA-A, HLA-B, and HLA-C proteins which are expressed on the surface of all nucleated cells. HLA class I molecule can bind viral peptides and present them on the surface of virus-infected cells, which can be recognized by CD8+CTL. While HLA class II molecule can bind and present viral peptides as well, the cells can be recognized by CD4+ T lymphocytes. Both HLA class I and HLA class II are found to be associated with the outcomes of HBV infection [11, 17, 18]. Some HLA class I molecules can function as ligands of natural killer (NK) cell receptors and become important regulators for NK cell [19, 20] and CD8+ T cell [21, 22]. Association studies on the outcomes of other viral infections, such as HCV [23], HIV [24, 25], human papillomavirus [26], and *Cytomegalovirus* (CMV) [27], supported the important role of HLA I when it functioned as ligands for Killer cell immunoglobulin-like receptor (KIR). Gao et al. observed that some combinations of KIR and specific HLA-C gene were correlated with the occurrence of hepatitis B [28]. We also reported that the polymorphisms of HLA class I and KIR could influence the development of HBV-associated liver cancer [29]. To investigate whether HLA I molecules as KIR ligands could influence the HBsAg clearance in this cohort, we grouped the HLA-A, HLA-B, and HLA-C alleles into three KIR ligand categories: *Bw4*, HLA-C group 1, and HLA-C group 2 as described [30], and then compared their frequencies between the persistent infection and HBsAg clearance groups. However, no association was observed (data not shown).

The relationship between HLA class I polymorphisms and the outcome of HBV infection has been explored previously, but this relationship does not appear to be universal since the investigated populations are different. In Taiwanese Aborigines, HLA-A*0206 was susceptible to HBV infection and chronicity, and HLA-B*4001 was likely to be associated with the elimination of the virus [10]. In American Caucasians, HLA-A*01-B*08-DRB1*03, B*44-Cw1601, and B*44-Cw*0501 haplotypes were associated with viral persistence, and HLA-A*0301 was associated with viral clearance. In European Caucasians, HLA-B*8 was associated with non-response to HBV vaccination [11]. Karan et al., in their studies in Turkey, have reported that *HLA-A24* and *CW1* were protective against chronic HBV [31]. Besides that, in eastern Turkey, *HLA-B35* and *HLA-CW4* were significantly higher in the chronic HBV group than those in the spontaneously recovered group [32].

In this study, we investigated a cohort of Qidong Han population who lived in an area of high prevalence of HBV infection. The samples were made of unrelated males and were followed up for 10 years. No previous prospective cohort study has been conducted to clarify the relationship between the polymorphisms of HLA alleles and the outcome of HBV infection in such a population. We found that HLA-A*33:03:01G was more frequent in the HBV persistent infection group than in the HBsAg clearance group (6.36% versus 2.86%, *P* = 0.025, *OR* = 2.31, 95% CI: 1.09–4.90), which was consistent with the results found in northern Iran [33] and in Korean population [34] although in these populations, the difference did not reach statistically significant level after correction.

The frequency of HLA-B*13:01:01G was significantly higher in the HBsAg clearance group than that in the persistent group (8.57% versus 3.46%, *P* = 0.0004, *OR* = 2.62, 95% CI: 1.51–4.54), which revealed that HLA-B*13:01:01G was associated with a protective effect against persistent HBV infection in this Qidong Han population. HLA-B*13:01:01G is not a *KIR *ligand because it does not carry a *Bw4 *motif. Therefore, it is unlikely that NK cell can function through this *KIR-HLA* interaction. Interestingly, the frequencies of HLA-B*13:02:01G were almost identical in the two groups (2.86% in HBsAg clearance group versus 3.46% in persistent infection group). HLA-B*13:02:01G differs from HLA-B*13:01:01G for only three amino acids at positions 94, 95, and 97. This three-amino-acid difference probably affects the conformation of the MHC binding grooves and results in their abilities to present viral peptide to CD8+ CTLs, which leads to different prognosis after HBV infection. This possible molecular mechanism needs to be tested in the future study.

In summary, we found that HLA-B*13:01:01G was associated with a protective effect against persistent HBV infection in Qidong Han population. Our study provides a new clue about the influence of HLA class I diversity on the natural history of HBV infection in this population. These findings suggest that the host HLA polymorphism is an important factor in determining the outcome of HBV infection. Studies of the underlying molecular mechanisms need to be carried out in the future.

## Figures and Tables

**Table 1:** Frequencies of HLA-A alleles in each study group.

HLA-A alleles	Persistent infection group (2*n* = 896)		HBsAg clearance group (2*n* = 280)	
	Allele frequency (%)	Count	Allele frequency (%)	Count
A*01:17	0.11	1	0.00	0
A*01:01:01G	2.00	18	2.86	8
A*02:01:25	0.00	0	0.36	1
A*02:03:01G	1.34	12	1.43	4
A*02:06:01G	6.58	59	5.71	16
A*02:07:01G	9.93	89	9.64	27
A*02:10	0.33	3	0.00	0
A*02:11:01G	0.22	2	0.36	1
A*02:53N	0.11	1	0.00	0
A*02:01:01G	15.29	137	16.79	47
A*03:01:01G	0.33	3	0.71	2
A*11:01:01G	18.30	164	21.07	59
A*11:02:01G	4.35	39	5.71	16
A*23:01:01G	0.00	0	0.36	1
A*24:04	0.11	1	0.36	1
A*24:07	0.33	3	0.00	0
A*24:08	0.11	1	0.00	0
A*24:10	0.11	1	0.00	0
A*24:20	0.33	3	0.71	2
A*24:77	0.00	0	0.36	1
A*24:02:01G	22.32	200	20.36	57
A*26:01:01G	2.57	23	2.14	6
A*29:01:01G	0.22	2	0.00	0
A*30:01:01G	2.23	20	1.43	4
A*31:01:02G	5.25	47	5.00	14
A*32:01:01G	0.22	2	0.36	1
A*33:11	0.11	1	0.71	2
A*33:03:01G*	6.36	57	2.86	8
A*34:01:01	0.11	1	0.00	0
A*68:01:02G	0.45	4	0.00	0
A*69:01	0.22	2	0.71	2

*P = 0.025 (Pc = 0.0017, so A*33:03:01G does not reach statistically significant after Bonferroni correction).

**Table 2:** Frequencies of HLA-B alleles in each study group.

HLA-B alleles	Persistent infection group (2*n* = 896)		HBsAg clearance group (2*n* = 280)	
	Allele frequency (%)	Count	Allele frequency (%)	Count
B*07:02:01G	0.11	1	0.00	0
B*08:01:01G	0.22	2	0.00	0
B*08:18	0.11	1	0.00	0
B*13:01:01G*	3.46	31	8.57	24
B*13:02:01G	3.46	31	2.86	8
B*13:06	0.11	1	0.00	0
B*15:02:01G	2.90	26	2.5	7
B*15:03:01G	0.11	1	0.00	0
B*15:07:01G	0.22	2	0.36	1
B*15:11:01G	3.24	29	4.26	12
B*15:12:01G	0.47	4	0.00	0
B*15:15	0.00	0	0.36	1
B*15:18:01G	1.34	12	1.43	4
B*15:25:01G	0.22	2	0.71	2
B*15:27:01	2.01	18	2.14	6
B*15:58	0.45	4	0.00	0
B*15:01:01G	12.05	108	10	28
B*18:02	0.11	1	0.00	0
B*18:01:01G	0.22	2	0.00	0
B*27:04:01G	4.35	39	4.29	12
B*27:05:02G	0.59	5	0.71	2
B*27:06	0.11	1	0.00	0
B*35:10	0.11	1	0.00	0
B*35:72	0.11	1	0.00	0
B*35:01:01G	0.59	5	0.71	2
B*37:01:01G	0.33	3	1.79	5
B*38:02:01G	0.45	4	0.00	0
B*39:01:01G	0.33	3	1.07	3
B*40:02:01G	3.57	32	3.57	10
B*40:03	0.33	3	0.00	0
B*40:06:01G	3.01	27	3.57	10
B*40:11:01	0.33	3	0.00	0
B*40:20	0.11	1	0.00	0
B*40:21	0.11	1	0.00	0
B*40:40	1.11	10	0.00	0
B*40:01:01G	16.52	148	15.36	43
B*41:01	0.00	0	0.36	1
B*44:03:01G	0.33	3	0.00	0
B*44:03:02	0.11	1	0.00	0
B*46:01:01G	18.30	164	16.43	46
B*48:01:01G	0.33	3	0.71	2
B*51:02:01	0.33	3	0.00	0
B*51:01:01G	1.00	9	0.36	1
B*52:01:01G	0.11	1	1.07	3
B*52:08	0.11	1	0.00	0
B*54:01:01G	6.03	54	7.14	20
B*55:02:01G	5.58	50	4.64	13
B*55:12	0.22	2	0.36	1
B*56:01:01G	0.78	7	0.36	1
B*56:03	0.22	2	0.00	0
B*57:01:01G	2.23	20	2.86	8
B*58:01:01G	0.59	5	0.36	1
B*59:01:01G	0.70	6	1.07	3
B*67:01:01	0.22	2	0.00	0

*P = 0.0004 (*P* < Pc. It is statistically significant after the Bonferroni correction).

**Table 3:** Frequencies of HLA-C alleles in each study group.

HLA-C alleles	Persistent infection group (2*n* = 896)		HBsAg clearance group (2*n* = 280)	
	Allele frequency (%)	Count	Allele frequency (%)	Count
C*01:02:01G	26.00	233	26.07	73
C*01:02:03	0.11	1	0.36	1
C*01:03:01G	1.00	9	1.07	3
C*03:02:01G	4.58	41	3.21	9
C*03:04:01G	11.83	106	14.29	40
C*03:04:04	0.00	0	0.71	2
C*03:32	0.33	3	0.00	0
C*03:69	0.11	1	0.00	0
C*03:03:01G	8.26	74	9.29	26
C*04:03	0.22	2	0.36	1
C*04:01:01G	6.92	62	3.93	11
C*06:02:01G	4.47	40	4.29	12
C*06:06	0.11	1	0.00	0
C*07:02:04	0.11	1	0.00	0
C*07:04:01G	0.89	8	1.07	3
C*07:08	0.22	2	0.00	0
C*07:27:01	0.22	2	0.00	0
C*07:43	0.33	3	0.00	0
C*07:51	0.11	1	0.00	0
C*07:56:01	0.11	1	0.36	1
C*07:01:01G	0.11	1	0.00	0
C*07:02:01G	12.72	114	13.93	39
C*08:03:01	0.22	2	0.36	1
C*08:01:01G	6.47	58	7.86	22
C*12:02:01G	4.91	44	5.00	14
C*12:03:01G	0.56	5	0.00	0
C*14:02:01G	7.14	64	4.29	12
C*14:03	0.11	1	0.00	0
C*15:02:01G	1.34	12	2.14	6
C*15:08	0.11	1	0.00	0
C*15:15	0.00	0	0.36	1
C*15:17	0.22	2	0.00	0
C*16:02:01G	0.11	1	0.71	2
C*17:01:01G	0.00	0	0.36	1
